# Performance-Enhanced Fiber-Optic Hydrogen Sensing Method Based on a Pd-Cu Alloy Microcantilever and a Reflective Enhancement Structure

**DOI:** 10.3390/s26144449

**Published:** 2026-07-13

**Authors:** Qiang Wang, Qiongxin Wu, Yajun Jia, Junjie Jiang, Zhijian Jin, Jiwei Du

**Affiliations:** 1 School of Electrical Engineering, Shanghai Jiao Tong University, Shanghai 200240, China; wqee-insulation@sjtu.edu.cn (Q.W.);; 2 Baotou Power Supply Branch, Inner Mongolia Electric Power (Group) Co., Ltd., Baotou 014000, China; 3 School of Electrical Engineering, Shanghai Dianji University, Shanghai 201306, China

**Keywords:** fiber-optic hydrogen sensor, Fabry–Perot interferometer, Pd-Cu alloy, microcantilever, reflective enhancement structure

## Abstract

To address the demand for early hydrogen monitoring in power equipment insulation systems, a fiber-optic Fabry–Perot (F-P) hydrogen sensor based on a Pd–Cu alloy microcantilever is proposed. The microcantilever serves as the force-sensitive structure, with a Pd–Cu alloy film deposited as the hydrogen-sensitive layer and an Au reflective layer introduced to enhance optical reflection and suppress thermal drift. Hydrogen absorption induces volume expansion of the Pd–Cu film, causing cantilever bending and a consequent variation in the F-P cavity length, which leads to a shift in the characteristic wavelength of the reflected spectrum and enables wavelength-demodulated hydrogen detection. Finite element analysis was conducted to investigate the stress distribution and displacement response, confirming the effective amplification effect of the microcantilever structure. Sensor fabrication, packaging, and hydrogen response experiments were subsequently carried out. The results show a good linear response in the hydrogen concentration range of 0–300 ppm, with a wavelength sensitivity of approximately 20.8 pm/ppm and a limit of detection of 3.24 ppm. In a 24 h stability test, the baseline fluctuation standard deviation was 22.46 pm, indicating good stability and repeatable sensing performance. Temperature variation produced a wavelength sensitivity of approximately 0.2734 nm/°C, and environmental condition tests further demonstrated stable operation under temperature, humidity, vibration, and electromagnetic disturbances. The proposed sensor exhibits immunity to electromagnetic interference, intrinsic safety, and compatibility with miniaturized integration, showing promising potential for low-concentration hydrogen monitoring in power equipment.

## 1. Introduction

Hydrogen is one of the earliest and most representative fault gases generated during the degradation of oil-immersed electrical equipment. In transformer bushings, transformers, and other high-voltage insulation systems, insulation defects such as partial discharge, local overheating, and low-energy electrical discharges can induce decomposition of insulating oil and cellulose, producing dissolved hydrogen before significant thermal or electrical failure becomes apparent. Owing to its high diffusion coefficient and low generation threshold, hydrogen usually appears earlier than hydrocarbon gases and is therefore widely regarded as an effective indicator for incipient fault diagnosis in dissolved gas analysis (DGA). Consequently, accurate detection of trace hydrogen has become an important approach for condition assessment and predictive maintenance of power equipment, contributing to improved operational reliability and reduced maintenance costs [1,2,3].

Unlike conventional hydrogen monitoring in open atmospheric environments, hydrogen detection in oil-immersed power equipment presents several unique challenges. Hydrogen is initially dissolved in insulating oil and subsequently diffuses toward the sensing interface, resulting in relatively slow mass transport. Meanwhile, practical operating environments are often accompanied by elevated temperatures, dissolved oxygen, moisture, electromagnetic interference, and other fault-related gases, all of which may influence hydrogen adsorption kinetics, sensing stability, and long-term reliability. Therefore, hydrogen sensors intended for transformer condition monitoring should not only exhibit high sensitivity and low detection limits, but also possess excellent electrical insulation, electromagnetic immunity, environmental adaptability, and long-term operational stability under complex service conditions [4,5].

To meet these requirements, various hydrogen sensing technologies have been extensively investigated, including electrochemical, semiconductor, catalytic combustion, and optical sensing methods. Electrochemical sensors generally provide high sensitivity and low power consumption; however, their performance is susceptible to electrolyte aging and humidity. Semiconductor sensors are compatible with MEMS fabrication and feature compact structures, but they often require elevated operating temperatures and may suffer from cross-sensitivity to reducing gases. Catalytic combustion sensors exhibit broad detection ranges but involve electrical heating elements that are less desirable for high-voltage insulation systems. By comparison, optical hydrogen sensors have attracted increasing attention owing to their intrinsic electrical insulation, immunity to electromagnetic interference, remote interrogation capability, and compatibility with compact fiber-optic integration. These advantages make optical sensing particularly suitable for dissolved hydrogen monitoring in high-voltage power equipment. Recent studies have demonstrated continuous progress in optical hydrogen sensing based on fiber gratings, Fabry–Perot interferometers, surface plasmon resonance, and other photonic structures, achieving improved sensitivity, stability, and detection limits [6,7,8,9,10,11,12].

Among various optical hydrogen sensing materials, Pd-based thin films remain the most widely adopted owing to their excellent catalytic dissociation and reversible hydrogen absorption characteristics. Nevertheless, pure Pd films generally suffer from hydrogen-induced phase transition, hysteresis, and long-term structural degradation. Alloying Pd with Cu effectively suppresses the α–β phase transition, alleviates hydrogen embrittlement, improves cyclic stability, and broadens the linear sensing range. In addition, MEMS microcantilever structures can efficiently convert hydrogen-induced surface stress into measurable mechanical deformation, while Fabry–Perot interferometric demodulation enables highly sensitive optical readout with excellent immunity to electromagnetic interference. Based on these considerations, this work proposes a fiber-optic Fabry–Perot hydrogen sensor integrating a Pd–Cu alloy microcantilever and an Au reflective enhancement structure. Finite-element simulation, device fabrication, sensor packaging, and hydrogen sensing experiments are systematically carried out to evaluate the sensing mechanism and practical applicability of the proposed device for transformer insulation monitoring [13,14,15,16,17]. It should be emphasized that the proposed sensor is primarily designed for controlled or temperature-compensated environments. For deployment in real substation conditions, additional temperature monitoring and signal compensation techniques are required to ensure measurement reliability under dynamic thermal fluctuations.

## 2. Principle and Fabrication

### 2.1. Principle of the Fabry–Perot Interferometric Cavity

**Figure 1:**
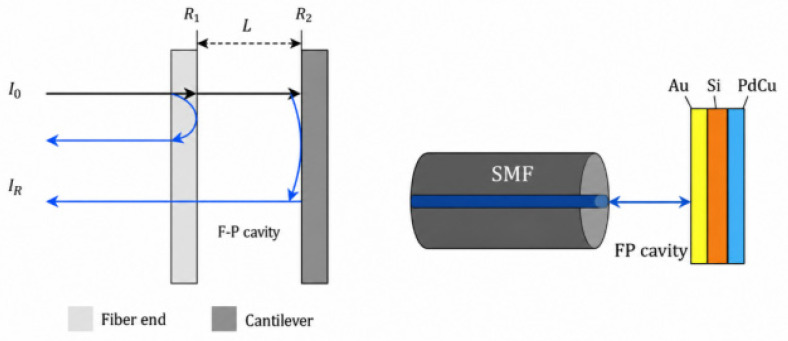
Principle of the Fabry–Perot (F-P) interferometric cavity and the structure of the interferometric cavity designed in this paper.

The main text of the article should appear here with headings as appropriate. As shown in Figure 1, a Fabry–Perot (F-P) interferometric structure consists of two parallel reflecting surfaces, and interference occurs when light undergoes multiple reflections between these two surfaces. In this study, the F-P cavity is formed by the end face of a single-mode optical fiber and the reflective surface of a MEMS cantilever. When incident light exits from the fiber end face, one part is reflected at the fiber end face, while the other part is transmitted into the air cavity and reflected back by the cantilever surface. These two reflected beams interfere at the fiber end face, and the interference intensity is related to the F-P cavity length [18,19]. The interference intensity can be expressed as

(1) $$I=I_{1}+{\;I}_{2\;}+2\sqrt{I_{1}I_{2}}\cos\left(\frac{4\pi \mathrm{nL}}{\lambda }\right)$$

where L is the F-P cavity length, λ is the optical wavelength, and n is the refractive index.

When the cantilever deforms, the F-P cavity length changes accordingly, resulting in a shift in the interference spectrum. The Pd–Cu alloy deposited on the cantilever surface exhibits good hydrogen absorption capability. When hydrogen molecules dissociate and adsorb on the Pd–Cu alloy surface, palladium hydride-related solid solution forms and induces lattice volume expansion. Owing to the thermal expansion mismatch between the sensitive layer and the silicon cantilever, this volume variation generates stress in the cantilever structure and consequently causes cantilever bending.

According to Sieverts’ law, the dissolved hydrogen concentration in palladium is proportional to the square root of the hydrogen partial pressure [20,21]:

(2) $$P_{H_{2}}^{1/2}\;=\;KX_{H_{2}}$$

where K is the Sieverts coefficient, which is a constant. At room temperature, its value is 350 Torr^1/2^, i.e., K = 46,662.7 Pa^1/2^. Under low hydrogen concentration conditions, assuming that the strain induced in pure palladium is ε, the initial palladium film length is l, and the deformation is Δl, the relationship with hydrogen concentration can be expressed as

(3) $$\varepsilon =\frac{\Delta l}{l}=0.026x_{H_{2}}$$

Combining the above equations, the deformation of palladium after hydrogen absorption under low hydrogen concentration can be written as:

(4) $$\Delta l\;=\;\frac{0.026IP_{H_{2}}^{1/2}}{K}$$

Compared with pure palladium, Pd–Cu alloys exhibit significant overall advantages in hydrogen sensing applications, mainly due to the synergistic regulation of the electronic structure and phase stability through alloying. First, the introduction of Cu suppresses the α-to-β hydride phase transition of pure Pd in a hydrogen atmosphere, thereby alleviating the hydrogen embrittlement phenomenon caused by abrupt lattice volume expansion and significantly improving the strain-fatigue resistance and long-term service stability of the sensing material. Second, the ligand effect and lattice compression effect introduced by alloying optimize the d-band center of Pd, which not only moderately lowers the dissociative adsorption energy barrier of hydrogen on the Pd surface to enable low-temperature response, but also broadens the linear response range of the sensor to hydrogen concentration by modulating the diffusion barrier of hydrogen atoms, while accelerating the response–recovery kinetics [21,22]. The Pd–Cu alloy used in this work has an atomic ratio of Pd:Cu = 7:3, which was selected to improve hydrogen-response stability while reducing the hysteresis typically associated with pure Pd films.

To improve the optical readout quality of the sensor and suppress the additional drift caused by ambient temperature fluctuations, an Au thin film was introduced on the backside of the silicon cantilever. On the one hand, this Au layer serves as a highly reflective interface facing the end face of the single-mode optical fiber, replacing the bare silicon surface in the Fabry–Perot interference process. As a result, the reflectivity of the second reflecting surface of the cavity is effectively increased, the returned optical intensity and interference signal strength are enhanced, and the signal-to-noise ratio as well as the peak/valley identification stability of the reflected spectrum are improved [23].

On the other hand, the Au layer and the PdCu sensitive layer are located on opposite sides of the silicon substrate. Under temperature variation, the thermal expansion mismatch of these two layers with respect to the silicon substrate induces thermally driven bending in opposite directions. Therefore, by properly designing the thickness matching relationship between the Au layer and the PdCu layer, first-order compensation of the thermally induced cantilever deflection can be achieved to some extent, thereby reducing the additional influence of temperature variation on the Fabry–Perot cavity length and the output wavelength. Thus, the backside Au film not only plays a role in reflection enhancement, but also contributes to temperature compensation, which is beneficial for improving the spectral demodulation stability and environmental adaptability of the sensor [23].

(5) $$\frac{h_{\mathrm{Au}}}{h_{\mathrm{PdCu}}}=\frac{E_{\mathrm{PdCu}}(\alpha_{\mathrm{Si}}-\alpha_{\mathrm{PdCu}})}{E_{\mathrm{Au}}(\alpha_{\mathrm{Si}}{-\;\alpha }_{\mathrm{Au}})}$$

As indicated by Equation (5), where $h_{\mathrm{Au}}$ is the thickness of the Au film, $h_{\mathrm{PdCu}}$ is the thickness of the PdCu film, as; $\alpha_{\mathrm{Si}}$ is the thermal expansion coefficient of silicon, $\alpha_{\mathrm{Au}}$ is the thermal expansion coefficient of gold, $\alpha_{\mathrm{PdCu}}$ is the thermal expansion coefficient of PdCu, $E_{\mathrm{Au}}$ is the Young’s modulus of gold, and $E_{\mathrm{PdCu}}$ is the Young’s modulus of PdCu, substitution of the actual parameter values shows that when the thickness ratio between the Au film and the PdCu film is around 1.4, the Au reflective layer provides partial thermal drift compensation by introducing an opposite-direction stress relative to the Pd–Cu sensing layer, thereby reducing but not fully eliminating temperature-induced cavity variations. However, a residual temperature dependence still exists, which necessitates external calibration in practical applications.

The sensor consists of a single-mode fiber end face and a MEMS cantilever, which together form a Fabry–Perot interferometric cavity. The single-mode fiber end face serves as the first reflecting surface, while the Au reflective layer on the cantilever surface serves as the second reflecting surface, with an air cavity formed between them. When hydrogen is absorbed by the PdCu sensitive layer, the cantilever undergoes slight bending, thereby changing the F-P cavity length and causing a shift in the interference spectrum. The cantilever is made of single-crystal silicon with structural dimensions of 2.5 mm in length, 600 μm in width, and 10 μm in thickness. A 70 nm Au film is deposited on the backside of the cantilever as the reflective layer, while a 50 nm PdCu alloy film is deposited on the front side as the hydrogen-sensitive layer. An air cavity of approximately 80 μm is formed between the single-mode fiber end face and the cantilever.

### 2.2. Simulation Analysis

In order to better account for the complexity of practical application environments, Pd–Cu alloy was selected as the hydrogen-sensitive layer in the simulation. The process by which Pd–Cu alloy absorbs hydrogen and induces strain in the cantilever can be described as follows. First, hydrogen molecules diffuse to the surface of the Pd–Cu alloy film and dissociate into hydrogen atoms under the catalytic action of palladium. This process follows the Langmuir adsorption model, in which the adsorption rate is proportional to the hydrogen partial pressure. Subsequently, the hydrogen atoms are inserted into the interstitial sites of the PdCu lattice to form a solid solution, denoted as PdCuHx_xx. As the hydrogen atoms occupy the interstitial sites, the lattice expands [21,22], and the expansion ratio β can be expressed as:

(6) $$\beta \;=\;\frac{\Delta V\;}{V_{0}}\;=\;k\cdot c$$

where c is the hydrogen atom concentration and k is the expansion coefficient, which is approximately 2.6 × 10^−3^ at −1 for the PdCu alloy.

The PdCu thin film with a thickness of 50 nm is deposited on the surface of a silicon cantilever with a thickness of 10 um. Since the silicon substrate constrains the free expansion of the thin film, compressive stress o is generated in the film. According to the bimaterial beam theory, the stress magnitude can be expressed as

(7) $$\sigma =E_{f}\cdot \beta \cdot c\cdot \frac{1}{1+\frac{E_{f}\;t_{f}}{E_{s}\;t_{s}}}$$

where Ef and Es are the Young’s moduli of the film and the substrate, respectively, with Ef = 100 GPa for PdCu and Es = 170 GPa for silicon; tf and ts, are the thicknesses of the film and substrate, respectively, i.e., 50 nm and 10 um. To distinguish the strain energy response of the two cantilever configurations under otherwise identical conditions, a hydrogen concentration of 100 ppm, which is representative for low-concentration hydrogen sensing, was selected for the simulation. At a given temperature, the hydrogen concentration is first converted into the hydrogen partial pressure $P_{H_{2}}$,. Then, the hydrogen solubility C in the alloy film is determined according to Sieverts’ law:

(8) $$c\;=\;K\sqrt{P_{H_{2}}}$$

where K is the solubility coefficient. This equation provides the fundamental basis for the feasibility of the simulation, and all subsequent simulations were carried out on this basis.

By combining the above relationships, the key expression for converting gas concentration into equivalent stress can be obtained as

(9) $$\sigma ={\;E}_{f}\cdot k\cdot K\sqrt{P_{H_{2}}}\cdot \frac{1}{1+\frac{E_{f}\;t_{f}}{E_{s}\;t_{s}}}$$

**Figure 2:**
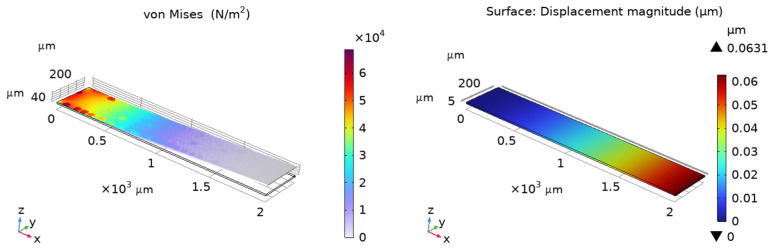
Stress distribution and displacement of the cantilever after hydrogen adsorption.

This equation provides the fundamental basis for the feasibility of the simulation, and all subsequent simulations were carried out on this basis. First, the equivalent load generated by hydrogen absorption at a given concentration was calculated according to the above equations. For the selected hydrogen concentration of 100 ppm, the equivalent applied load was determined to be 1 MPa based on Equation (9) together with engineering compensation factors from relevant references. In COMSOL Multiphysics 6.1, a uniform load of 1 MPa was applied to the cantilever, and the steady-state deformation of the cantilever under one-end-fixed boundary condition was analyzed. As shown in Figure 2, hydrogen absorption induces lattice expansion in the Pd-based film, resulting in a gradient internal stress field within the cantilever. The maximum stress is concentrated near the fixed end constraint region and decays approximately along the beam length. This internal stress drives the cantilever to form a linearly distributed displacement field, with the displacement gradually increasing from the fixed end to the free end. The maximum displacement at the free end reaches 63.1 nm, which verifies the sensing mechanism from hydrogen absorption to stress generation and further to displacement output, thereby providing a theoretical basis for sensor structure optimization and sensitivity enhancement [24].

According to Equation (10), the cantilever displacement obtained from the simulation can then be converted into the corresponding spectral shift measured by the optical spectrum analyzer:

(10) $$\Delta \lambda \;\approx \;-\lambda \cdot \frac{\delta }{d}$$

where Δλ is the spectral shift, λ is the reference wavelength of the interference spectrum, δ is the maximum free-end displacement of the cantilever, and d is the length of the Fabry–Perot cavity.

### 2.3. Sensor Fabrication

**Figure 3:**
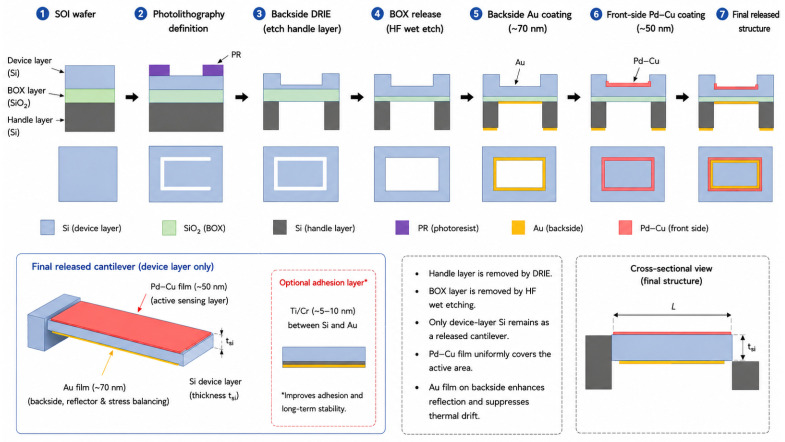
Schematic diagram of sensor fabrication.

The MEMS cantilever structure was fabricated using standard microelectromechanical systems (MEMS) processing technology, and the detailed fabrication procedure is illustrated in Figure 3. First, a silicon-on-insulator (SOI) wafer (Simgui Technology Co., Ltd., Shanghai, China) consisting of a silicon device layer, a buried oxide (BOX) layer, and a silicon handle layer was selected as the starting substrate. The cantilever pattern was defined on the device layer by conventional photolithography. Subsequently, backside deep reactive ion etching (DRIE) (Oxford Instruments PlasmaPro 100 Cobra, Oxford Instruments Plc, Abingdon, UK) was performed to remove the silicon handle layer beneath the cantilever region until the BOX layer was exposed. The exposed BOX layer was then selectively removed by hydrofluoric acid (HF) wet etching to release the cantilever, leaving only the silicon device layer as the suspended MEMS beam. After the cantilever was released, an Au thin film(Ningbo Jiangfeng Electronic Materials Co., Ltd., Ningbo, China) with a thickness of approximately 70 nm was deposited on the backside of the cantilever by electron-beam evaporation to serve as the reflective layer, thereby enhancing the optical reflectivity of the Fabry–Pérot cavity while introducing stress balancing to suppress thermal drift. Finally, a Pd–Cu alloy thin film (Ningbo Jiangfeng Electronic Materials Co., Ltd., Ningbo, China) with a thickness of approximately 50 nm was deposited onto the active region on the front side of the cantilever as the hydrogen-sensitive layer. The Pd–Cu film uniformly covers the sensing area, allowing the hydrogen-induced surface stress to be efficiently transferred to the released silicon cantilever, thereby producing measurable mechanical deflection for optical hydrogen sensing [19,22].

**Figure 4:**
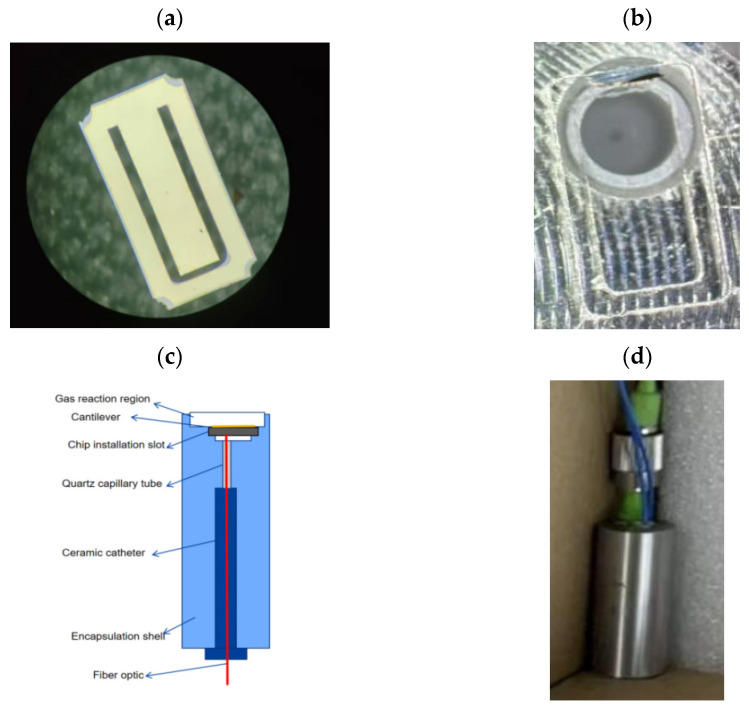
Schematic diagram of sensor packaging (**a**) MEMS cantilever chip, (**b**) Packaging groove, (**c**) Sensor packaging schematic, (**d**) Packaged sensor probe.

To improve the mechanical stability of the MEMS cantilever sensor and achieve stable coupling between the optical fiber and the sensitive structure, a dedicated packaging design was developed for the MEMS cantilever chip. Figure 4 shows the packaging structure and the actual packaged device. As shown in Figure 4a, the MEMS cantilever chip under a microscope serves as the hydrogen-sensitive element, with the hydrogen-sensitive material deposited on its surface for hydrogen concentration detection. To protect the microstructure and facilitate engineering application of the sensor, a stainless-steel packaging structure was designed. As shown in Figure 4b, a packaging groove was machined in the stainless-steel substrate for placement of the MEMS chip. This groove enables accurate chip positioning and provides sufficient space for adhesive fixation. After the MEMS cantilever chip was placed into the groove, epoxy adhesive was used to fix the chip, thereby ensuring mechanical stability. Figure 4c presents the schematic diagram of the sensor packaging structure. The single-mode optical fiber was aligned along the axial direction of the stainless-steel package with the cantilever structure, and an optical sensing cavity was formed between the fiber end face and the cantilever surface. Figure 4d shows the actual packaged sensor probe. The cylindrical stainless-steel housing not only effectively protects the MEMS microstructure but also improves the stability and reliability of the sensor in practical application environments [19].

**Figure 5:**
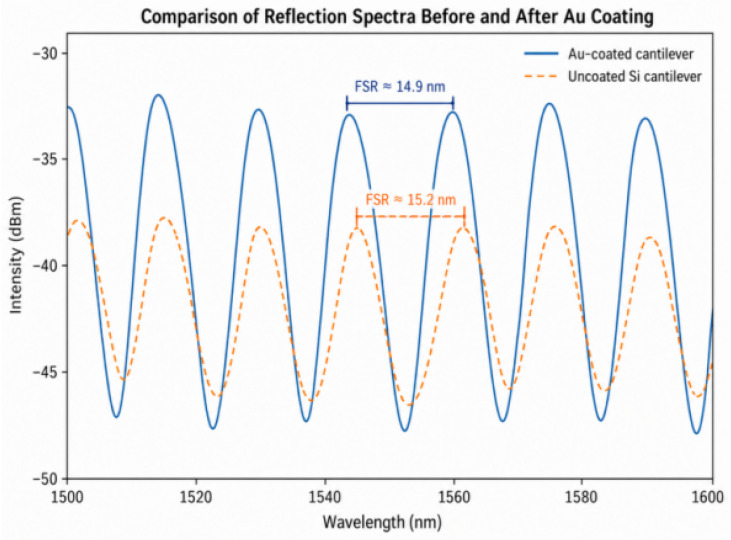
Initial output spectra of air chamber FP sensing probes with and without gold-plated films.

According to the initial output spectrum of the air-cavity F-P sensor probe shown in Figure 5, the free spectral range (FSR) was measured to be approximately 14.9 nm. For a low-finesse Fabry–Perot interferometric cavity, the FSR satisfies

(11) $$\mathrm{FSR}\;\approx \;\frac{\lambda^{2}}{2\mathrm{nL}}$$

where λ is the central wavelength, n is the refractive index inside the cavity, and L is the cavity length. By taking λ= 1550 nm, n = 1 for air, and FSR = 14.9 nm, the initial cavity length formed by the hydrogen-sensitive cantilever and the fiber end face was calculated to be approximately 80.6 μm.

As can be seen from the comparison spectra, both the Au-coated cantilever and the uncoated silicon cantilever were able to generate periodic Fabry–Perot interference spectra under otherwise similar conditions, and their free spectral ranges remained close to each other. This indicates that the equivalent cavity lengths of the two structures were generally consistent, while slight deviations in FSR arose from practical assembly errors and differences in the reflecting interfaces. In comparison, the reflected spectrum of the Au-coated cantilever exhibited a significantly higher overall optical intensity than that of the uncoated silicon cantilever, manifested by an upward shift of the average reflected intensity and more pronounced peak-to-valley variation, indicating a substantial reflection-enhancement effect. This is because, after the introduction of the Au thin film as a highly reflective interface, the reflectivity of the second reflecting surface of the cavity became higher than that of the bare silicon surface, thereby increasing the returned optical power and enhancing the absolute modulation amplitude of the interference signal. Although the uncoated silicon cantilever could also produce interference fringes, its effective surface reflectivity was lower, leading to a weaker overall reflected intensity and a smaller peak-to-valley difference. As a result, the demodulation process for the uncoated silicon cantilever was more susceptible to source fluctuation, noise disturbance, and spectral peak identification error.

The advantage of the Au-coated cantilever is not limited to the increase in reflected intensity, but also includes improved spectral demodulation stability. For the uncoated silicon cantilever, the relatively weak returned optical signal leads to a lower signal-to-noise ratio at the optical spectrum analyzer. Under low hydrogen concentration conditions, the small wavelength shift induced by hydrogen absorption can therefore be more easily buried in background noise, reducing the peak-tracking accuracy. In contrast, after Au coating, the interference spectrum becomes more distinguishable, and the characteristic peak and valley positions become clearer, which is beneficial for improving wavelength demodulation resolution and repeatability. In addition, the Au thin film can also act as a compensating layer on the backside of the cantilever. Together with the PdCu sensitive layer on the upper surface, it forms a dual-film structure that generates thermally induced bending in opposite directions under temperature variation, thereby suppressing temperature drift to some extent. Therefore, the use of an Au-coated cantilever not only improves the reflected optical power and the quality of the F-P interference signal, but also enhances the sensor stability, anti-interference capability, and environmental adaptability under low-concentration hydrogen detection conditions.

## 3. Results and Discussion

### 3.1. Hydrogen Sensing Experimental Platform

To investigate the hydrogen sensing performance of the proposed optical sensor, the sensor probe was placed in a gas reaction chamber. The hydrogen concentration in the mixed gas was controlled by adjusting the flow-rate ratio of two mass flow controllers, while the total flow rate of the gas mixture was fixed at 2 L/min. The pressure inside the sealed reaction chamber was maintained constant, and the ambient temperature was set at 25 °C. First, nitrogen was introduced into the chamber to establish a stable baseline condition. Subsequently, a hydrogen–nitrogen gas mixture (Air Liquide China Holding Co., Ltd., Shanghai, China) with a specified concentration was continuously introduced into the chamber for 10 min.

**Figure 6:**
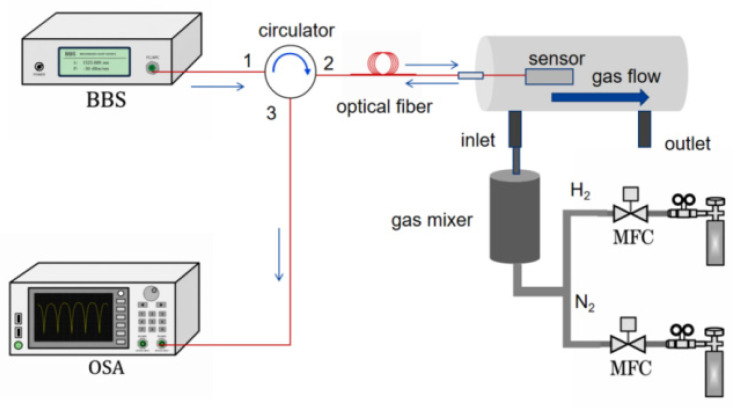
Schematic diagram of hydrogen sensing performance verification.

The experimental platform used for hydrogen sensing validation is shown in Figure 6. The system mainly consisted of a broadband light source (Shenzhen Hoyatek Technology Co., Ltd., Shenzhen, China), an optical spectrum analyzer (AQ6370D Yokogawa Test & Measurement Corporation, Tokyo, Japan), the fabricated fiber-optic F-P hydrogen sensor probe, a sealed gas chamber, and a gas mixing unit composed of mass flow controllers (Beijing Sevenstar Electronics Co., Ltd., Beijing, China). During the experiment, the mixed gas entered the reaction chamber and interacted with the Pd–Cu sensitive layer deposited on the cantilever surface. The corresponding cavity-length variation induced by cantilever deflection was then monitored in real time through the reflected interference spectrum. By controlling the hydrogen concentration and recording the spectral evolution, the hydrogen response characteristics of the sensor could be systematically evaluated.

### 3.2. Spectral Response to Hydrogen

**Figure 7:**
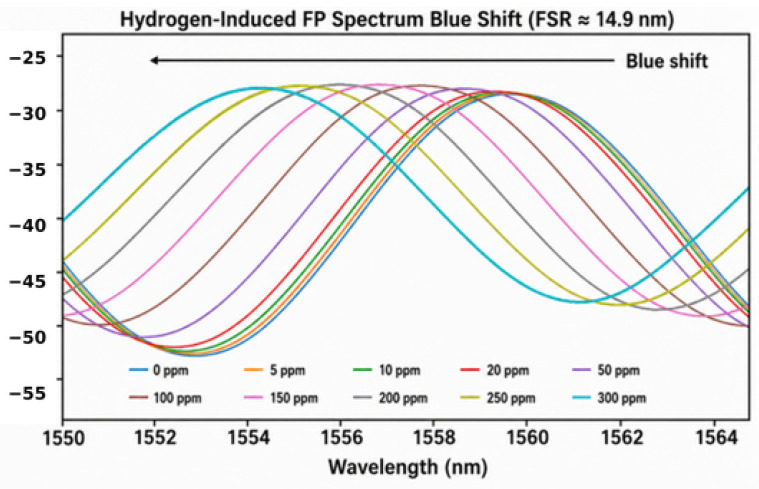
Variation in reflection spectra at different hydrogen concentrations.

As shown in Figure 7, hydrogen absorption by the Pd–Cu alloy film caused deformation of the sputtered sensitive layer and resulted in a decrease in the air-cavity length. Consequently, the reflected spectrum shifted toward the shorter-wavelength direction with increasing hydrogen concentration. The investigated hydrogen concentration range was selected as 0–300 ppm, mainly to match the gas concentration level relevant to transformer bushing insulating oil. At relatively low concentrations, smaller concentration intervals were adopted in order to preliminarily determine the detection limit of the designed sensor and to compare it with the limit subsequently derived from the baseline noise standard deviation. From the experimental results, the total spectral shift at 300 ppm hydrogen was approximately 6.24 nm, corresponding to a hydrogen sensitivity of 20.8 pm/ppm.

Meanwhile, based on the simulation results presented in the previous section, the free-end displacement of the microcantilever under 100 ppm hydrogen was approximately 63.1 nm. According to Equation (10), this displacement can be converted into a spectral shift of about 1.23 nm under the simulated conditions, whereas the experimentally measured wavelength shift at 100 ppm hydrogen was 1.71 nm. Although a certain discrepancy exists between the simulated and experimental values, both results remain within the same order of magnitude, and the deviation is relatively limited. This indicates that the proposed simulation model is capable of reasonably describing the sensing chain of “hydrogen absorption—thin-film stress variation—cantilever deflection—spectral wavelength shift,” and can therefore reflect the actual response behavior of the device to a considerable extent.

It should also be noted that, in the current simulation process, the hydrogen adsorption, dissolution, and stress transfer processes were simplified to a certain degree. As a result, some deviation between the simulation and experimental results is unavoidable. Nevertheless, the simulation provides an effective reference for structural parameter design and dimensional optimization, and partially compensates for the difficulty of directly and accurately mapping hydrogen absorption behavior to the equivalent displacement load of the cantilever. In future work, by further incorporating more realistic material parameters, interfacial stress transfer characteristics, and packaging boundary conditions into the simulation model, better agreement between the predicted results and the experimentally measured performance is expected, which would in turn provide a more reliable theoretical basis for structural optimization of the sensor.

### 3.3. Dynamic Response and Recovery Characteristics

**Figure 8:**
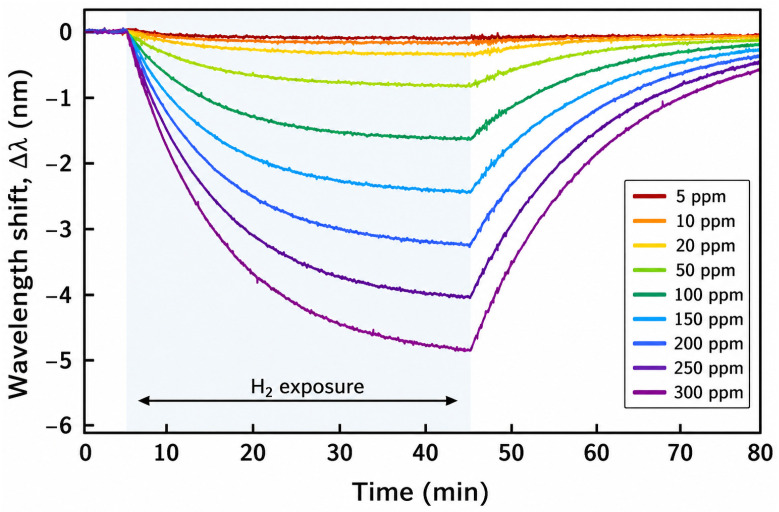
Time-response characteristics at different hydrogen concentrations.

In addition to spectral sensitivity, the response time is another important performance indicator. The dynamic response characteristics of the proposed hydrogen sensor are shown in Figure 8. In this work, the response time is defined as the time interval from the onset of spectral peak shift to the final stabilization of the wavelength under hydrogen exposure, while the recovery time is defined as the time required for the spectrum to return toward its initial position and reach a stable state after the hydrogen concentration has become stable and the gas is subsequently released.

As shown in Figure 8, the response curves under different hydrogen concentrations are plotted together. The measured response times fall within the range of approximately 45–50 min. The relatively long response time is mainly attributed to the fact that, after the gas is introduced into the reaction chamber, a certain period is required for the gas concentration inside the chamber to reach a stable equilibrium. In addition, hydrogen molecules entering the predefined reaction region are first adsorbed by the cantilever, which occupies part of the available adsorption sites and gradually slows down the subsequent adsorption process, especially under low gas-flow and low-concentration conditions. At lower hydrogen concentrations, the response amplitude is smaller, whereas the time required to reach full stabilization is shorter than that at higher concentrations.

A similar trend can also be observed in the recovery process. In the present work, natural desorption was adopted during the recovery stage, namely, the two valves of the reaction chamber were opened to release the gas. Under such conditions, the hydrogen desorption process from the cantilever is relatively slower than the adsorption process. This behavior can be attributed to several factors. First, hydrogen storage and trap sites exist within the thin film, resulting in slow hydrogen release. Second, at low hydrogen concentrations, the residual hydrogen partial pressure inside the reaction chamber cannot be rapidly reduced to zero, thereby weakening the driving force for dehydrogenation. Third, since the cantilever operates as a stress–deformation output structure, a certain degree of mechanical recovery lag is also involved. Finally, under low-signal conditions, the tail end of the recovery process approaches the noise floor, which further prolongs the apparent recovery time [21,24]. The response time in this work is governed by gas dissolution, diffusion, and adsorption processes under oil-immersed conditions. Therefore, the observed time scale is suitable for long-term monitoring of slow-evolving incipient faults in power equipment, rather than transient or rapid gas-evolution events.

### 3.4. Linearity and Repeatability

**Figure 9:**
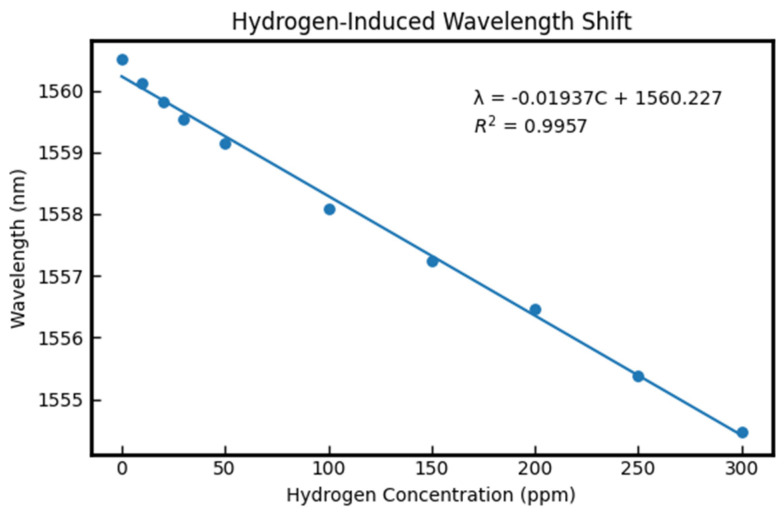
Fitting curves for different hydrogen concentrations.

Figure 9 presents the linear fitting results of the wavelength response under different hydrogen concentrations. The average hydrogen sensitivity was approximately 19.37 pm/ppm. It can also be seen that the rate of wavelength variation with hydrogen concentration is relatively higher at low concentrations than at high concentrations. This behavior mainly suggests a certain degree of nonlinearity in the hydrogen response. According to Equation (9), the cantilever displacement exhibits an approximately square-root dependence on hydrogen concentration, while the spectral shift is approximately proportional to the cantilever displacement. In addition, practical imperfections in the Fabry–Perot cavity may also contribute to the reduction in linearity. Nevertheless, based on the obtained fitting coefficient, it can still be concluded that the wavelength shift maintains a strong linear correspondence with hydrogen concentration within the low-concentration range considered in this study [20,21,22].

**Figure 10:**
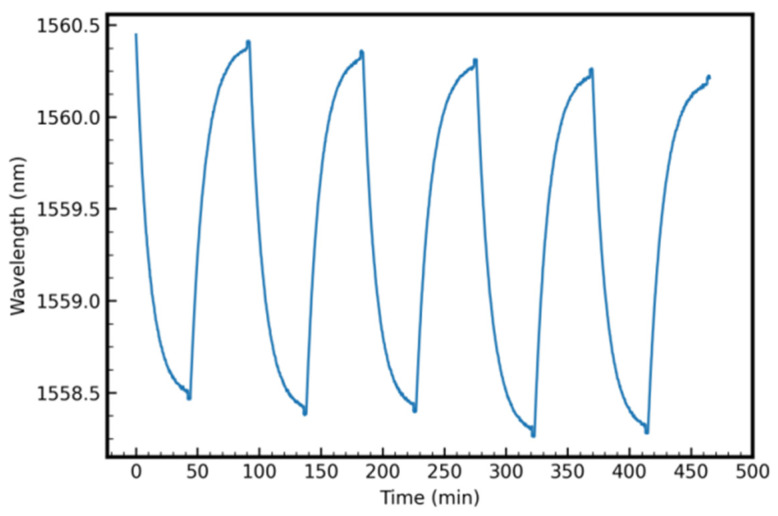
Hydrogen repeatability test.

Repeatability is also an important criterion in sensor performance evaluation. Figure 10 shows the cyclic hydrogen charging and discharging experiment conducted at a hydrogen concentration of 100 ppm. It can be observed that the response and recovery trends remain generally consistent during repeated measurements, and the wavelength shifts all stay within approximately ±10% of the value predicted by the above fitting relationship. However, the recovered initial wavelength gradually decreases in the subsequent cycles. This phenomenon is mainly attributed to interfacial stress relaxation and hysteresis in the Pd–Cu thin film during repeated hydrogen adsorption–desorption cycles, rather than structural failure or delamination of the sensing layer. Therefore, the observed baseline drift corresponds to a transition between reversible and quasi-stable states. Even so, the recovery remains within an acceptable range for normal sensing operation. These results indicate that the sensor maintains a reasonable response consistency under repeated exposure, although its anti-aging performance and expected service lifetime still need to be further evaluated. In addition, periodic zero-point recalibration is recommended during data conversion and long-term operation, which may help alleviate performance degradation and zero drift under conditions involving severe concentration variation [25]. In practical applications, this drift can be corrected through periodic zero-point calibration or baseline re-normalization to ensure long-term measurement stability.

### 3.5. Temperature Response and Compensation Analysis

The key sensing structure of the F-P cavity is formed by the end face of the single-mode optical fiber positioned inside the package and the cantilever surface aligned opposite to it. The package housing and the fiber-fixing component are mechanically stabilized by UV-curable adhesive. Therefore, when the ambient temperature changes, the packaging components and the cantilever materials, owing to their different thermal expansion coefficients and positional constraints, undergo relative axial displacement. This relative displacement changes the length of the air cavity and consequently causes a shift in the interference peak of the spectrum. Such spectral drift may be mistakenly interpreted as a variation in hydrogen concentration, thereby leading to false signals. For this reason, the structural design against temperature interference is indispensable for the sensor.

From the perspective of the cantilever itself, the substrate is made of silicon, while the two sides of the cantilever are coated with a Pd–Cu alloy film and an Au film, respectively. By properly designing the thickness ratio of these two films, the positional drift caused by the expansion and contraction of different materials can be partially balanced.

For the packaging part where the optical fiber is fixed, material selection was mainly considered in order to reduce relative displacement. In this work, Invar was adopted as the packaging material. Due to its extremely low coefficient of thermal expansion, the thermally induced dimensional change of Invar under ambient temperature fluctuation or chamber temperature rise is much smaller than that of ordinary metallic materials. This can effectively suppress the additional cavity-length variation caused by thermal expansion and contraction of the package, thereby reducing the risk of misinterpreting temperature disturbances as hydrogen response signals and improving the zero-point stability, measurement repeatability, and long-term reliability of the system. This advantage is particularly important for low-concentration hydrogen sensing, where the signal amplitude is relatively small and temperature-induced cross-sensitivity can more easily obscure the actual hydrogen response.

However, Invar also has certain limitations. For example, compared with some commonly used metallic materials, its thermal conductivity and machinability are not always advantageous, which may increase the fabrication difficulty and cost during microstructural packaging. Moreover, although the low thermal expansion characteristic of Invar can significantly reduce cavity-length drift induced by temperature variation, it still cannot completely eliminate the residual thermal effects caused by thermal stress mismatch among the cantilever films, the adhesive interface, and the optical fiber itself. Therefore, in high-precision applications, further improvement of temperature-interference resistance still requires additional temperature compensation strategies or structural optimization [26].

**Figure 11:**
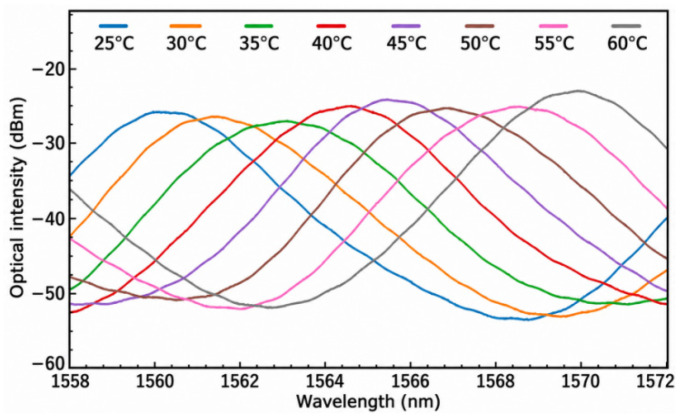
Sensor temperature response test.

**Figure 12:**
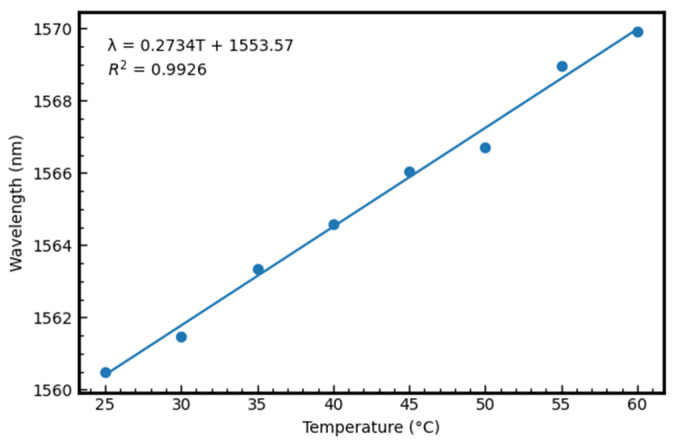
Fitting curve of wavelength versus temperature.

As shown in Figure 11, the temperature response of the designed sensor was investigated by monitoring the shift of the interference peak in the temperature range from 25 °C to 60 °C, with each temperature point maintained for 10 min. The fitting result presented in Figure 12 shows that the average temperature sensitivity reached 0.2734 nm/°C, with a correlation coefficient of 0.9926, indicating a clear linear relationship between wavelength variation and temperature. As the temperature increased, the materials on the cantilever expanded, while the Invar packaging structure also underwent nanometer-scale thermal expansion, causing an overall increase in the F-P cavity length and resulting in a redshift of the spectrum. Conversely, during cooling, the combined contraction of the cantilever materials and the Invar package reduced the cavity length and led to a blueshift of the spectrum. It should be noted that this temperature dependence originates from the combined thermo-mechanical response of the cantilever structure and packaging system, rather than an independent sensing channel. Therefore, the obtained temperature sensitivity represents the intrinsic cross-sensitivity of the Fabry–Perot structure.

In practical applications, this cross-sensitivity can be effectively mitigated by introducing an external temperature reference sensor or applying a calibration-based compensation model, where the hydrogen concentration is corrected using a wavelength–temperature coupling matrix.

It should be noted that the observed variation in reflected spectral intensity under temperature changes is mainly attributed to slight thermo-mechanical deformation of the MEMS cantilever, which slightly modifies the Fabry–Pérot cavity alignment without affecting the wavelength-demodulation accuracy. When the room temperature varied by 0.1 °C, the equivalent hydrogen concentration change of the sensor was approximately 1.30 ppm. This result indicates that, under properly controlled temperature conditions, the influence of temperature on hydrogen measurement accuracy can be reduced to a negligible level. Furthermore, for practical operating environments involving larger temperature fluctuations, numerical compensation may be performed according to the fitted temperature-response relationship, since real-time temperature monitoring systems are generally available in field applications. By incorporating such information into the signal-processing algorithm, the final hydrogen concentration output can be corrected in a timely manner [27]. Future work will focus on integrating a dual-channel sensing configuration to independently monitor temperature and hydrogen signals, enabling improved decoupling of thermal effects.

### 3.6. Long-Term Stability and Detection Limit

**Figure 13:**
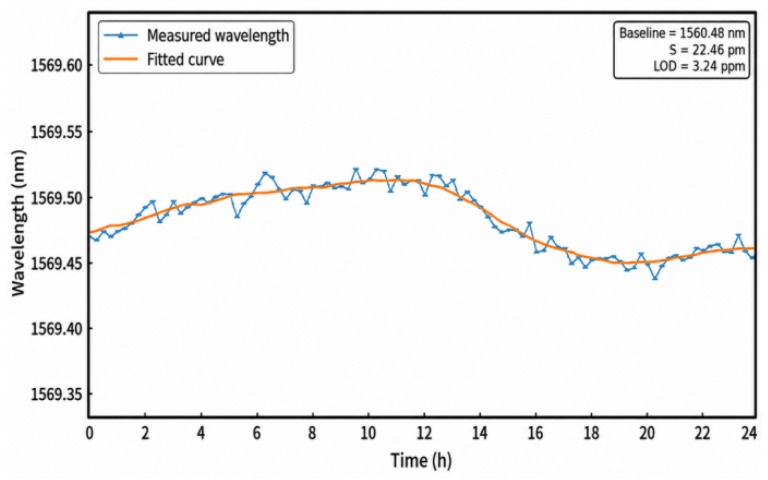
Fitting curve of wavelength drift within 24 h.

To evaluate the long-term baseline stability of the proposed fiber-optic MEMS hydrogen sensing system, the output wavelength of the sensor was continuously monitored for 24 h under a constant hydrogen-free background condition. As shown in Figure 13, the central wavelength remained around 1560.48 nm throughout the test, exhibiting only slow and small fluctuations, with an overall trend of first increasing slightly and then gradually decreasing. This low-frequency drift is consistent with weak diurnal variations in laboratory temperature, airflow, and instrument operating conditions, indicating that no abrupt jump or sustained instability occurred in the system output and that the sensor possesses good long-term operational stability.

Meanwhile, the deviation between the measured curve and the fitted curve remained small, suggesting that the wavelength fluctuation mainly originated from the superposition of low-level environmental disturbances and intrinsic system noise, rather than from structural failure of the sensing head or abnormal demodulation. This further verifies that the hydrogen sensor can provide stable and repeatable signal output under long-duration continuous monitoring conditions.

For quantitative analysis, statistical processing of the 24 h wavelength sequence yielded a baseline wavelength standard deviation of σ22.46 pm. Combined with the hydrogen sensitivity of 20.8 pm/ppm, the limit of detection (LOD) was calculated according to the expression LOD = 3σ/S, resulting in a value of approximately 3.24 ppm. This demonstrates that the sensor not only has good baseline stability, but is also capable of ppm-level low-concentration hydrogen detection, thus satisfying the basic requirements of trace hydrogen online monitoring in terms of both sensitivity and stability, and showing promising application potential in energy-storage safety monitoring and related hydrogen early-warning scenarios under complex environments [28].The key performance parameters of the gold-plated and unplated cantilever sensors are summarized in Table 1. As can be seen, the Au reflective layer improves the hydrogen responsivity and reduces the temperature sensitivity while maintaining good linearity.

### 3.7. Influence of Operating Conditions on Sensor Performance

To evaluate the applicability of the proposed sensor under practical power equipment operating environments, performance tests were conducted under different environmental conditions while maintaining a constant hydrogen concentration of 100 ppm and allowing stabilization under each individual condition. The investigated operating conditions included normal environment (25 °C, 50% RH), elevated temperature (80 °C), high humidity (85% RH), power-frequency vibration (100 Hz), and external magnetic field (500 μT), and the corresponding results are summarized in Table 2.

As shown in Table 2, under normal ambient conditions, the sensor exhibited an initial central wavelength of approximately 1560.48 nm, with a spectral response magnitude of 1.71 nm. The response and recovery times were 48 min and 52 min, respectively, while the baseline fluctuation remained within ±15 pm, indicating stable output performance and satisfactory measurement repeatability.

Under the elevated-temperature condition, the initial wavelength shifted to 1575.52 nm, corresponding to an approximately 15.04 nm redshift relative to room temperature. This phenomenon indicates that temperature variation affects the Fabry–Perot cavity length through thermal expansion of the structural materials and packaging components, thereby changing the spectral position. Meanwhile, the response time decreased to 35 min and the recovery time was shortened to 38 min, suggesting that increased temperature promotes hydrogen diffusion and accelerates the adsorption–desorption kinetics of the Pd–Cu alloy layer. However, the spectral response magnitude slightly decreased to 1.62 nm, representing a reduction of approximately 5.3% compared with that under room-temperature conditions. This behavior may be attributed to reduced effective stress conversion efficiency within the sensitive layer at elevated temperature. Nevertheless, the response degradation remained limited, indicating that the introduced Au compensation layer partially suppressed thermally induced additional deformation.

Compared with temperature effects, the high-humidity condition introduced more pronounced degradation in dynamic performance. Under 85% RH, the response and recovery times increased to 58 min and 64 min, respectively, while the spectral response magnitude decreased to 1.53 nm, corresponding to a reduction of approximately 10.5%. Meanwhile, the baseline fluctuation increased to ±22 pm. This behavior suggests that moisture may influence local gas transport and surface adsorption processes, thereby reducing the effective hydrogen partial pressure reaching the sensitive layer and introducing additional low-frequency disturbance. Despite this influence, the sensor maintained distinguishable wavelength responses, demonstrating acceptable robustness against humidity variation.

Under the power-frequency vibration condition (100 Hz), the sensor exhibited good mechanical stability. The spectral response magnitude remained at 1.70 nm, showing only a slight deviation of approximately 0.6% compared with the normal condition. The response and recovery times remained nearly unchanged, although the baseline fluctuation slightly increased to ±20 pm. These results indicate that the cantilever structure and packaging design effectively suppress vibration-induced interference and maintain stable Fabry–Perot cavity operation.

Under an external magnetic field of 500 μT, the sensor performance remained essentially unchanged compared with the normal condition. The initial wavelength, response time, and spectral response magnitude all showed negligible variation, and the spectral response remained at 1.71 nm with a baseline fluctuation of only ±16 pm. This result confirms that the optical wavelength demodulation mechanism is inherently immune to electromagnetic interference because no electrical signal acquisition is required in the sensing region. This characteristic represents an important advantage of fiber-optic sensing compared with conventional electrical gas sensors in high-voltage environments.

Overall, among the investigated environmental factors, temperature remains the dominant source affecting output stability, whereas humidity mainly influences response dynamics and baseline noise. In contrast, vibration and magnetic field have relatively limited influence on sensing performance. These results demonstrate that the proposed Pd–Cu microcantilever-based fiber-optic Fabry–Perot hydrogen sensor maintains stable operation under complex environmental conditions and exhibits promising potential for online dissolved hydrogen monitoring in transformer bushing insulation systems. Further improvement in long-term measurement accuracy may be achieved by integrating reference channels or compensation algorithms in future studies.

### 3.8. Comparison with State-of-the-Art Optical Hydrogen Sensors

To further evaluate the performance of the proposed sensor, a comparison with representative state-of-the-art optical hydrogen sensors reported in recent years is summarized in Table 3. As shown, most reported optical hydrogen sensors are primarily designed for gas-phase hydrogen detection and therefore exhibit relatively fast response times. In contrast, the proposed sensor is specifically developed for trace hydrogen monitoring in oil-immersed power equipment, where the sensing process is governed by hydrogen dissolution, diffusion, and adsorption in insulating oil. Although the response time is relatively longer, the proposed sensor demonstrates a competitive limit of detection (3.24 ppm) and high wavelength sensitivity (20.8 pm/ppm) within the low-concentration range of 0–300 ppm. Furthermore, the combination of a Pd–Cu alloy MEMS cantilever and a fiber-optic Fabry–Pérot interrogation structure provides intrinsic electromagnetic immunity, electrical insulation, and compact integration capability, making it well suited for long-term online monitoring of high-voltage power equipment.

## 4. Conclusions

In this work, a fiber-optic Fabry–Perot hydrogen sensor based on a Pd–Cu alloy microcantilever was designed and fabricated for low-concentration hydrogen monitoring in power equipment. The sensor utilizes the volume expansion of the Pd–Cu thin film upon hydrogen absorption to drive cantilever bending, and the resulting variation in the F-P cavity length causes a shift in the characteristic wavelength, enabling wavelength-demodulated hydrogen detection. Simulation results indicate that the designed microcantilever structure effectively amplifies the stress response of the sensitive thin film. Experimental results demonstrate a linear response to hydrogen in the range of 0–300 ppm, with a wavelength sensitivity of approximately 20.8 pm/ppm and a limit of detection of 3.24 ppm. The 24 h baseline test yielded a standard deviation of 22.46 pm, demonstrating good short-term stability and repeatable sensing performance. Temperature experiments reveal a certain degree of cross-sensitivity to ambient temperature, indicating that further improvement in measurement accuracy may be achieved through structural optimization, reference-channel introduction, or temperature-compensation algorithms. In addition, environmental condition evaluations verified that the proposed sensor maintained stable sensing characteristics under variations in temperature, humidity, vibration, and electromagnetic disturbances, indicating favorable environmental adaptability. Overall, the proposed Pd–Cu alloy microcantilever-based fiber-optic F-P hydrogen sensor combines the advantages of electromagnetic immunity, miniaturization, and remote interrogation capability, providing a feasible sensing solution for hydrogen monitoring and condition awareness in power equipment applications.

## Figures and Tables

**Table 1:** Comparison of sensing performance between gold-plated and unplated cantilevers.

Cantilever Type	Gold-Plated Cantilever	Unplated Cantilever
Hydrogen responsivity/(pm·ppm^−1^)	20.8	16.2
Wavelength shift at 100 ppm/nm	1.71	1.36
Linear fitting R^2^	0.994	0.987
Temperature sensitivity/(nm·°C^−1^)	0.2734	0.3580
Average reflected light intensity/dBm	−34.8	−38.9
FSR/nm	14.9	15.1

**Table 2:** Comparison of Key Sensor Performances under Different Operating Conditions (100 ppm H_2_, independent stabilization under each operating condition).

Operating Conditions	Initial Peak Wavelength (nm)	Response Time (min)	Recovery Time (min)	Spectral Response Magnitude (nm)	Baseline Fluctuation (pm)
Normal Environment (25 °C, 50% RH)	1560.48	48	52	1.71	±15
High Temperature 80 °C	1575.52	35	38	1.62	±18
High Humidity 85% RH	1560.51	58	64	1.53	±22
Power Frequency Vibration 100 Hz	1560.48	49	53	1.70	±20
Strong Magnetic Field 500 μT	1560.48	48	52	1.71	±16

**Table 3:** Comparison of representative optical hydrogen sensors reported in recent literature.

Sensor Structure	Sensitive Material	Detection Range	Sensitivity	LOD	Response Time	Main Features	Ref.
Fiber-optic Fabry–Pérot cavity with suspended membrane	Pd membrane	Low-concentration H_2_	3.6 pm/ppm	3.3 ppm	Not specified	High sensitivity and low LOD based on suspended Pd membrane	[29]
Fiber Bragg grating hydrogen sensor	Hydrogen-doped Pt/WO_3_	Not specified	184-fold improvement compared with pure Pt/WO_3_	30 ppm	25 s	Fast response and enhanced sensitivity	[30]
Optical fiber evanescent sensor	Pd nanoparticles on etched FBG	Gas-phase H_2_	Not specified	Not specified	<90 s	Short response and no obvious hysteresis	[31]
Fiber-optic Fabry–Pérot hydrogen sensor	Pd-decorated graphene	Gas-phase H_2_	Not specified	~20 ppm	18 s	Compact structure and fast response	[32]
Temperature-compensated fiber-optic hydrogen sensor	PdNi-coated Fabry–Pérot cavity	1% H_2_	1383 pm at 1% H_2_	Not specified	16 s	Temperature-compensated dual-cavity design	[33]
This work	Pd–Cu alloy MEMS microcantilever with Au reflective layer	0–300 ppm	20.8 pm/ppm	3.24 ppm	45–50 min	Low-concentration detection, MEMS Fabry–Pérot structure, electromagnetic immunity, compact fiber-optic integration	—

## Data Availability

No new data were created or analyzed in this study.
